# The Exploration of Joint Toxicity and Associated Mechanisms of Primary Microplastics and Methamphetamine in Zebrafish Larvae

**DOI:** 10.3390/toxics12010064

**Published:** 2024-01-12

**Authors:** Hao Wang, Jindong Xu, Yang Yuan, Zhenglu Wang, Wenjing Zhang, Jiana Li

**Affiliations:** 1 College of Oceanography, Hohai University, Nanjing 210098, China; 15725388264@163.com (H.W.); aukuma@gmail.com (J.X.); yy15252540515@126.com (Y.Y.); 15854287023@163.com (W.Z.); 2 West China School of Public Health, West China Fourth Hospital Sichuan University, Chengdu 610041, China; m18018567955@163.com; 3 Ningbo Academy of Ecological, Environmental Sciences, Ningbo 315000, China

**Keywords:** microplastics, methamphetamine, joint toxicity, zebrafish, health

## Abstract

The co-existence of microplastics (MPs) and methamphetamine (METH) in aquatic ecosystems has been widely reported; however, the joint toxicity and associated mechanisms remain unclear. Here, zebrafish larvae were exposed individually or jointly to polystyrene (PS) and polyvinyl chloride (PVC) MPs (20 mg/L) and METH (1 and 5 mg/L) for 10 days. The mortality, behavioral functions, and histopathology of fish from different groups were determined. PS MPs posed a stronger lethal risk to fish than PVC MPs, while the addition of METH at 5 mg/L significantly increased mortality. Obvious deposition of MPs was observed in the larvae’s intestinal tract in the exposure groups. Meanwhile, treatment with MPs induced intestinal deposits and intestinal hydrops in the fish, and this effect was enhanced with the addition of METH. Furthermore, MPs significantly suppressed the locomotor activation of zebrafish larvae, showing extended immobility duration and lower velocity. METH stimulated the outcome of PS but had no effect on the fish exposed to PVC. However, combined exposure to MPs and METH significantly increased the turn angle, which declined in individual MP exposure groups. RNA sequencing and gene quantitative analysis demonstrated that exposure to PS MPs and METH activated the MAPK signaling pathway and the C-type lectin signaling pathway of fish, while joint exposure to PVC MPs and METH stimulated steroid hormone synthesis pathways and the C-type lectin signaling pathway in zebrafish, contributing to cellular apoptosis and immune responses. This study contributes to the understanding of the joint toxicity of microplastics and pharmaceuticals to zebrafish, highlighting the significance of mitigating microplastic pollution to preserve the health of aquatic organisms and human beings.

## 1. Introduction

Microplastics (MPs) typically refer to plastic particles with a size (longest dimension of the particle) of less than 5 mm, which take the form of microspheres, fibers, and fragments [1]. Considered an emerging pollutant, microplastics undergo a succession of physical, chemical, and biological processes, ultimately permeating the environment, including rivers, oceans, lakes, and soil [2,3]. Due to their wide distribution, resistance to degradation, affinity for adsorbing environmental pollutants, and ease of entering organisms, the ubiquity of microplastics in aquatic ecosystems might pose a threat to both ecological and human health [4,5]. Some scholars examined the microplastic pollution in Turkey’s water environments and found that there was microplastic pollution in all kinds of these environments in the country [6]. Given their small size, MPs can be mistaken for food and ingested by zooplankton, fish, and crustaceans [7]. Hence, microplastics have been detected within various organisms, including mammals, birds, mollusks, and fish [8,9], further indicating the associated environmental risks. Previous studies have shown that exposure to microplastics could induce physical damage, inhibit physiological and biochemical functions, alter behavioral patterns, and reduce reproductive capacity and survival rates of organisms [10,11]. Studies have shown that rainbow trout exposed to polyethylene can exhibit growth inhibition, tissue damage, and cell apoptosis [12]. In addition, ingestion of microplastics by fish can lead to tissue oxidative stress and inhibit enzyme activity [13]. At present, controlling microplastic pollution is very urgent. Research has found that commercial feed provided during fish growth can also lead to a certain degree of microplastic pollution in various fish products [14]. Among the different types of MPs, the predominant ones in the environment were polypropylene (PP), polystyrene (PS), and polyvinyl chloride (PVC) [15]. PVC is one of the most produced commodity plastics globally, being extensively used in various human activities [16]. Some studies have also shown that PVC exhibits certain acute toxicity, as large copepods exposed to PVC leachates displayed signs of toxicity [17]. African catfish (*Clarias galliepinus*) exposed to PVC particles can exhibit adverse reactions such as neurotoxicity and oxidative stress [18]. Similarly, PS is one of the most commonly produced plastics globally [19], with research revealing that PS microbeads can accumulate in sea urchin intestines and negatively affect their embryonic development [20]. Research has also found that ingestion of PS particles can cause damage to the liver and gills of rainbow trout, resulting in negative impacts on their health [21]. Scholars have also found that polystyrene microplastics can weaken the predator-induced defenses of *Daphnia*, which may have a certain impact on aquatic ecology [22]. Although many studies have investigated the toxicity of PS or PVC to aquatic animals, the differences in toxicity induced in aquatic species by PS and PVC are so far unknown.

Besides the effect of single MPs, it is well known that MPs have a strong absorption capacity for organic chemicals [23]. Therefore, the co-existence of MPs and pollutants creates combined toxicity to organisms [24]. For example, MPs antagonized the toxicity of microalgae induced by triclosan [25] while enhancing the adverse effects on algae caused by procainamide [26]. A study has found that when polyethylene microplastics are combined with acetochlor, PE MPs significantly enhance the acute toxicity of ACT to zebrafish. In addition, it also increases the accumulation of ACT in zebrafish and exacerbates the oxidative stress damage of ACT in the intestine [27]. Scholars have conducted joint exposure of microplastics and phenanthrene to marine medaka (*Oryzias melastigma*), and the results indicate that the interaction between MPs and Phe may have a significant impact on the gut microbiota and metabolism of aquatic organisms [28]. Hence, the combined toxicity of MPs and emerging organic pollutants on aquatic organisms should be a concern.

Methamphetamine (METH) is a synthetic drug commonly used as a stimulant and hallucinogen [29]. Recently, it has become a prevalent illicit drug due to its highly addictive nature [30]. Similar to traditional pharmaceuticals, METH cannot be metabolized completely and thus enters the environment in the form of parent and metabolites [31]. The toxic effects and dependency mechanisms of METH are associated with central nervous system signaling pathways, the dopaminergic system, neurotransmitters, and molecular genetics [32]. Considering the conservation of the central nervous signaling pathways between vertebrates, exposure to METH might interact with dopamine, norepinephrine, and serotonin receptors in fish [32], showing a stimulating response [33]. Additionally, it has been revealed that METH can induce neuronal apoptosis, autophagy, and behavioral sensitization [34,35,36], causing oxidative stress and neuroinflammation [37,38]. The presence of METH in water environments might pose hazards to aquatic organisms and ecosystems. For example, research has indicated that the presence of METH in water interferes with the respiration, metabolism, and reproductive functions of aquatic organisms, leading to death and growth retardation [39]. The ecology-associated behaviors and processes of aquatic organisms (i.e., elegans) were disrupted by METH at low levels (50 ng/L), showing ecological system outcomes [40]. A previous study has found that the adsorption of METH by microplastics could lead to increased METH ingestion by aquatic organisms [41]. Therefore, the combined effects of METH with MPs in aquatic environments are highly likely to pose an increased threat to aquatic organisms.

This study aims to elucidate the combined toxicity of PVC and PS with METH by using zebrafish as an animal model. Zebrafish was selected as the model organism because it is a well-established model for assessing the toxicity of various substances [42]. The indicators, including mortality, behavioral functions, and histopathology of zebrafish in different groups, were determined. Furthermore, transcriptomic profiles of fish exposed to MPs and MPs combined with METH were established, and the enriched genes were quantitatively analyzed. Accordingly, the underlying toxicity mechanisms were elucidated.

## 2. Materials and Methods

### 2.1. Experimental Animals and Materials

Zebrafish embryos were obtained from Shanghai FeiXi Biotechnology Co., Ltd. (Shanghai, China) and incubated at the Marine Science Experimental Center, Hohai University. The animal research protocol was approved by the Institutional Animal Care and Use Committee. PVC and PS MPs powders (average diameter 10 μm) were purchased from Dongguan Junxin Plastics Co., Ltd. (Dongguan, China). The purity and characteristics of microplastics powders were identified by using a Thermo Scientific Nicolet iN10 infrared spectrometer (Waltham, MA, USA) (Figure S1), and particle size distribution was measured by using a Malvern Mastersizer 2000 laser particle size analyzer (Akribis Scientific Limited, Cheshire, UK) (Figure S2).

Zebrafish embryos (24 h post fertilization) were randomly placed in a fresh 60 mm Petri dish (20 each) spiked with embryo-rearing media (ERM; containing H_2_O, NaCl, CaCl_2_, KCl, MgSO_4_, pH 7.2). After hatching, zebrafish larvae were transferred to new Petri dishes for further cultivation, maintaining strict water quality and environmental conditions with dissolved oxygen > 7.0 mg/L, pH 7.0~7.4, no residual chlorine, ammonia, or nitrite. The light cycle was set at 14:10, the cultivation temperature was maintained at 26 ± 1 °C, and the larvae were fed twice daily with fresh brine shrimp nauplii. METH hydrochloride was purchased from Cerilliant Corporation (Round Rock, TX, USA), and a stock solution (1 mg/mL) was prepared by using deionized water and diluted with ERM as needed. Three replicates for one exposure concentration were set: (1) control; (2) 20 mg/L PS MPs; (3) 20 mg/L PS MPs + 1 mg/L METH (PS + 1); (4) 20 mg/L PS MPs + 5 mg/L METH (PS + 5); (5) 20 mg/L PVC MPs; (6) 20 mg/L PVC MPs + 1 mg/L METH (PVC + 1); (7) 20 mg/L PVC MPs + 5 mg/L METH (PVC + 5). The concentrations we used were obtained from other acute exposure experiments in the literature [41,43,44,45,46,47]. The exposure experiment lasted for 10 days with daily renewal of the exposure solutions in each Petri dish.

### 2.2. Survival Rate Determination

During the exposure period, the morphology of each zebrafish larvae was daily observed by using a stereo microscope (Upshine Instrument Co., Ltd., Suzhou, China). The basis for determining the death of zebrafish juveniles was the observation of cardiac arrest under stereo microscope [48]. Survival and mortality rates of zebrafish larvae in each experimental group were recorded daily.

### 2.3. Behavioral Assessment

At the end of exposure, behavioral functions of zebrafish larvae were assayed (eleven replicates for each group, n = 11) according to a previous publication [49]. Briefly, the locomotor trajectory of zebrafish was recorded using a CCD camera installed at the top of the tank for a duration of 10 min after 30 min acclimation, then analyzed by using XT7 software (Noldus IT, Wageningen, The Netherlands) to obtain the total swimming data of fish [50]. When observing the movement trajectories of zebrafish larvae, each extracted larva was individually observed. The behavioral functional indicators analyzed were mean velocity (cm/s), total distance (cm), turn angle, and immobility duration (%).

### 2.4. Histopathological Examination

The zebrafish were sacrificed by being rapidly cooled down after the 10-day exposure. Considering the small size of the animal, the whole of each larva was fixed using 4% paraformaldehyde (PFA) solution for 24 h, followed by dehydration using a gradation of ethanol series (increasing concentrations from high to low), and then embedded in paraffin (five replicates for each group, n = 5). Cross sections of approximately 8 μm thickness were obtained from the paraffin blocks and stained with hematoxylin and eosin (H&E). The obtained tissue sections were analyzed for histopathological changes by using an optical microscope.

### 2.5. RNA Extraction, Sequencing, and Analysis

In order to identify pathways and genes affected by combined toxicity and better comply with the 3R principle, the total RNA of the zebrafish larvae in the control, PS, PS + 5, PVC, and PVC + 5 groups was extracted by Trizol (Invitrogen, Carlsbad, CA, USA) according to the manufacturer’s protocol (three replicates for each group). RNA purity was checked by using the NanoPhotometer^®^ spectrophotometer (IMPLEN, Calabasas, CA, USA), integrity was assessed by using the RNA Nano 6000 Assay Kit of the Agilent Bioanalyzer 2100 system (Agilent Technologies, Santa Clara, CA, USA), and concentration was measured by using the Qubit^®^ RNA Assay Kit in the Qubit^®^2.0 Fluorometer (Life Technologies, Carlsbad, CA, USA). Library construction and RNA sequencing were performed on an Illumina Novaseq platform. The detailed protocols are shown in the Supplementary Materials.

### 2.6. Quantitative Real-Time PCR Analysis

According to the results of transcriptomics analysis, seven genes (i.e., *srfa*, *elk1*, *elk2*, *jun*, *mapk9*, *nfatc2a*, and *nfatc4*) of the fish from the control, PS, and PS + 5 groups were relatively quantitatively analyzed, while six genes (i.e., *cyp3c1*, *cyp3c4*, *cyp19a1a*, *pla2g4ab*, *nfatc2a*, and *nfatc4*) of the fish from the control, PVC, and PVC + 5 groups were chosen as candidates for Q-RT-PCR analysis. The detailed protocols are described in the Supplementary Materials, and all primer sequences are shown in Table S1.

### 2.7. Statistical Analysis

The statistical program SPSS 18.0 (Chicago, IL, USA) was used to analyze all the collected data. Five or more replicates of each parameter were determined to eliminate the variability of the results. All the data are expressed as mean ± standard deviations (S.D.). One-way analysis of variance (ANOVA) was used to compare and analyze the differences in mortality rate and behavioral parameters. One-way ANOVA followed by Turkey’s test was used to determine the differences in gene transcription levels of zebrafish larvae between the exposure and control groups. Two-way ANOVA analysis was used to determine the differences in the temporal changes of mortality of fish in the different groups. The differences in the mortality rates of fish in different groups were calculated by using the χ^2^ test. A *p*-value of less than 0.05 (95% confidence interval) was considered statistically significant.

## 3. Results

### 3.1. The Mortality of Zebrafish Larvae Was Influenced by MPs or MPs and METH, and Histopathological Changes Occured in the Tissues

**Figure 1:**
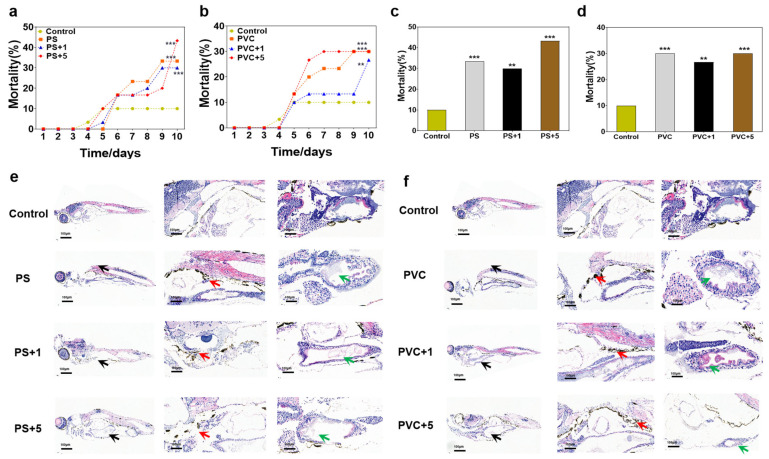
The mortality of zebrafish larvae was influenced by MPs or MPs and METH, and histopathological changes occurred in the tissues. The trajectory curves of mortality at different time points of PS, PS + 1, and PS + 5 (**a**). The trajectory curves of mortality at different time points of PVC, PVC + 1, and PVC + 5 (**b**). The terminal mortality of control, PS, PS + 1, and PS + 5 (**c**). The terminal mortality of control, PVC, PVC + 1, and PVC + 5 (**d**). ** *p* < 0.01, *** *p* < 0.001 vs. the control. Histopathological effects of zebrafish larvae after 10 days of exposure to MPs and METH (**e**,**f**). Abnormal morphology of enlarged trunk (black arrow), intestinal deposits (red arrows), and intestinal hydrops (green arrow).

There was no mortality observed among zebrafish larvae in both the control and exposure groups during the acclimation stage. As shown in Figure 1a, the mortality of fish in the PS exposure groups was significantly greater than in the control from the sixth day onward (*p* < 0.01), and the climbing speed in the PS + 5 group was much higher than in the other exposure groups. Similarly, the mortality in the PS groups was significantly greater than in the control from the fifth day, and the patterns of change in the PVC and PVC + 5 groups were parallel, showing steeper increases than in the PVC + 1 group (see Figure 1b). For the final mortality rates of different groups (Figure 1c,d), the values in the PS, PS + 1, and PS + 5 groups (33.3%, 30%, and 43.3%, respectively) were much higher than in the control (10%, *p* < 0.05), with the peak observed in the PS + 5 group (significantly greater than in the PS and PS + 1 groups). The absolute levels in the PVC (30%), PVC + 1 (26.7%), and PVC + 5 (30%) groups were markedly greater than in the control (10%, *p* < 0.05), with the peak in the PVC group (insignificant differences among the exposure groups).

According to the results of H&E staining of the whole body, obvious MP deposition and intestinal hydronephrosis were found in the fish in the exposure groups compared to the control (where the larvae displayed normal morphology, and no significant abnormalities were observed in the internal tissues). As shown in Figure 1e, the larvae in the PS exposure groups showed abnormal morphology compared to the control (black arrow), the front halves of juvenile fish trunks were found to become enlarged in the combined exposure groups. There were obvious particle depositions in the gastrointestinal tracts of fish (red arrow) in the exposure groups, with insignificant differences between the different PS groups. Moreover, an inflated enteric cavity was observed in the larvae from the exposure groups compared to the control (green arrow), indicating the occurrence of intestinal hydrops, showing a dose-dependent response. The changes in the PVC exposure groups showed similar patterns to the PS groups (Figure 1f).

### 3.2. The Behavioral Functions Were Affected by MPs or MPs and METH

**Figure 2:**
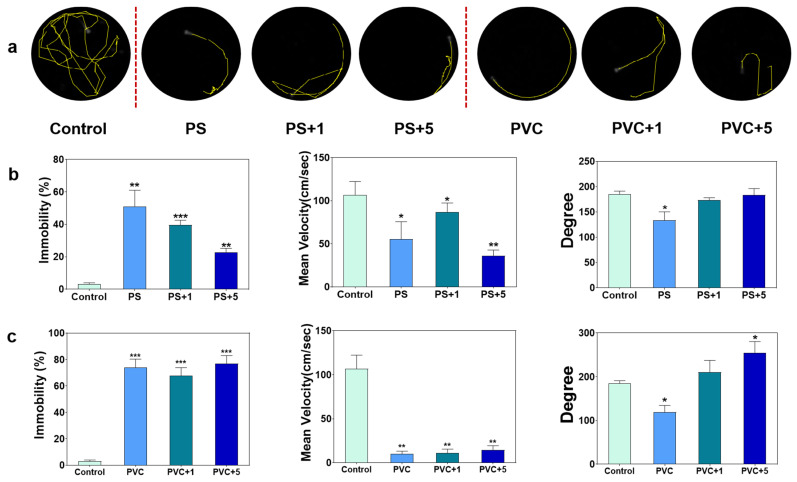
Behavioral changes in zebrafish larvae after 10 days of exposure to MPs and METH. Behavioral trajectories of zebrafish larvae after exposure, photographed from above (**a**). The mobility rate, mean velocity, and degree of zebrafish larvae of the group exposed to PS and METH (**b**). The mobility rate, mean velocity, and degree of zebrafish larvae of the group exposed to PVC and METH (**c**). * *p* < 0.05, ** *p* < 0.01, *** *p* < 0.001 vs. the control.

Through analysis of recorded movement trajectories of zebrafish larvae, it was observed that larvae in the control group exhibited exploratory swimming behavior characterized by long distances swam and strong randomness in swimming direction (Figure 2a). However, individual or combined exposure of MPs and METH significantly limited the locomotion of the larvae, which showed relatively simple swimming patterns in comparison with the control (Figure 2a). Statistically (Figure 2b,c), the immobility (%) of fish in the PS groups (51%, 39.5%, and 22.8%) was significantly higher than in the control group (3.2%). The combined exposure of PS and METH partially neutralized this effect, and the value of the PS + 5 group was significantly lower than that of the PS exposure group. Moreover, the mean velocity (cm/s) of the fish in the PS groups (55.3 cm/s, 86.7 cm/s, and 36.3 cm/s) declined markedly compared to the control group (106.9 cm/s), and there were no differences among the exposure groups. The individual exposure to PS decreased the turn angle of the larvae (*p* < 0.05), and the addition of METH ameliorated this change. For PVC groups, the immobility (%) of fish in the exposure groups (74.1%, 67.8%, and 77.1%) were markedly greater than the control group (3.2%), while the mean velocity (cm/sec) values were much lower (9.8 cm/s, 11.1 cm/s, and 14.3 cm/s), and no differences were found between different exposure groups. Notably, PVC individual treatment decreased the turn angle (118.9), but the addition of METH increased the turn angle of the fish, for which the value in the PVC + 5 (255.4) group was markedly higher than that in the control group (185.5).

### 3.3. The Gene Profiles of Zebrafish Larvae Were Affected by MPs or MPs and METH

**Figure 3:**
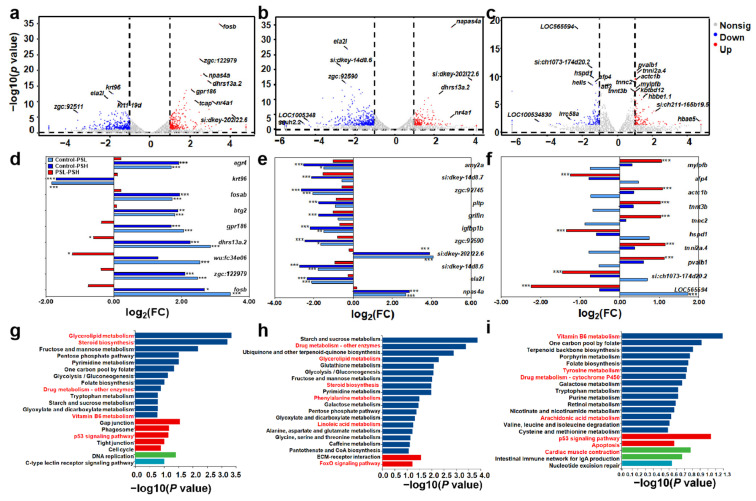
The gene profiles of zebrafish larvae were affected by PS or PS and METH. The differentially expressed genes (**a**–**c**). The top 30 DEGs (**d**–**f**). * *p* < 0.05, ** *p* < 0.01, *** *p* < 0.001. The enrichment pathways (**g**–**i**).

To elucidate the underlying molecular mechanisms of the adverse outcomes of zebrafish larvae induced by MPs or MPs + METH, RNA-seq was performed to establish the whole-genome expression profiling changes of fish in the control, PS, PS + METH, PVC, and PVC + METH groups. For the control and PS exposure group (Figure 3a), 551 differentially expressed genes (DEGs) were identified, comprising 226 upregulated genes and 325 downregulated genes, while there were 597 DEGs identified between the control and PS + 5 groups (175 upregulation and 422 downregulation, Figure 3b). Between the PS experimental group and the PS + 5 experimental group (Figure 3c), 235 DEGs were enriched, with 110 genes upregulated and 125 genes downregulated.

Among the DEGs, there were some overlap genes, like the downregulated gene ela2l and the upregulated gene napas4a. For the top 30 DEGs (Figure 3d–f), most genes from the control–PS and control–PS + 5 groups changed with consistent patterns, but they were different from the PS–PS + 5 groups, like *egr4*, *fosab*, *fosb*, *ela2l*, *npas4a*, and *btg2*. As a result, different pathways were enriched (Figure 3g–i). Between the control and exposure groups, drug metabolism and the P53 signaling pathway were enriched. For control–PS, glycerolipid metabolism, steroid biosynthesis, and vitamin B6 metabolism were significantly identified, while phenylalanine metabolism, linoleic acid metabolism, and the foxO signaling pathway were identified from the control–PS + 5 groups. Between the PS and PS + 5 groups, tyrosine metabolism, arachidonic acid metabolism, and cardiac muscle contraction were predominant. Based on the DEGs and the enrichment pathways, the correlation networks were established (Figure S3). The downregulated genes *cel.1* and *cel.2* were identified both in glycerolipid metabolism and steroid biosynthesis from the control–PS and control–PS + 5 groups, while the upregulated genes *rrm2* and *LOC100330664* involved in the P53 signaling pathway and drug metabolism were annotated. Meanwhile, the upregulated gene *plk2b* was linked to the FoxO signaling pathway. However, the genes involved in the P53 signaling pathway and drug metabolism were different from the PS and PS + 5 groups, *cycsb* and *aox5*, respectively.

**Figure 4:**
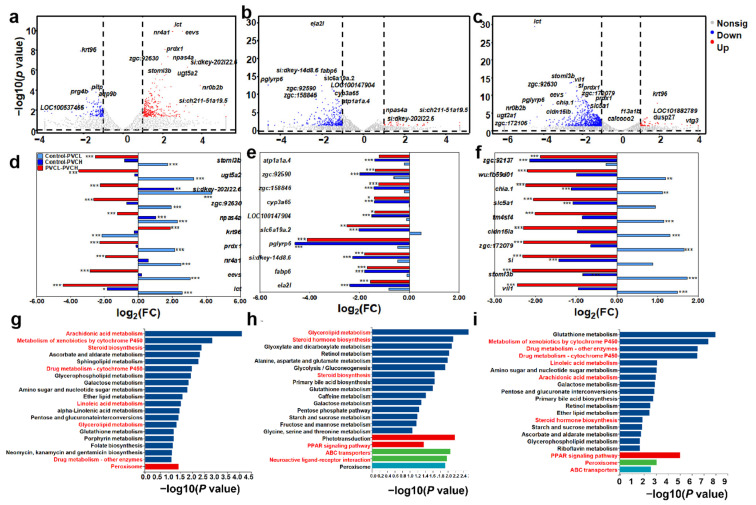
The gene profiles of zebrafish larvae were affected by PVC or PVC and METH. The differentially expressed genes (**a**–**c**).The top 30 DEGs (**d**–**f**). * *p* < 0.05, ** *p* < 0.01, *** *p* < 0.001. The enrichment pathways (**g**–**i**).

For the control and PVC exposure groups (Figure 4a), 427 differentially expressed genes (DEGs) were identified, comprising 285 upregulated genes and 142 downregulated genes, while there were 322 DEGs identified between the control and PVC + 5 groups (33 upregulation and 289 downregulation, Figure 4b). Between the PVC experimental group and the PVC + 5 experimental group (Figure 4c), 661 DEGs were enriched, with 51 genes upregulated and 610 genes downregulated.

Among the DEGs, there were some overlap genes, like the downregulated gene fabp6 and the upregulated gene napas4a. For the top 30 DEGs (Figure 4d–f), most genes from the control–PVC + 5 and PVC–PVC + 5 groups changed with consistent patterns, but they were different from the control–PVC groups, like *lct*, *cyp3a65*, *plyrp6*, *fabp6*, *npas4a*, and *si*. As a result, different pathways were enriched (Figure 4g–i). Between the control and exposure groups, glycerolipid metabolism and steroid biosynthesis were enriched. For the control–PVC groups, arachidonic acid metabolism, metabolism of xenobiotics by cytochrome P450, and peroxisome were significant pathways, while steroid hormone biosynthesis, the PPAR signaling pathway, and ABC transporter were identified from the control–PVC-5 group. Between the PVC and PVC-5 groups, metabolism of xenobiotics by cytochrome P450, linoleic acid metabolism, and peroxisome were predominant. Based on the DEGs and the enrichment pathways, the correlation networks were established (Figure S4). The downregulation gene *acsl5* was identified both in the PPAR signaling pathway and peroxisome from the control–PVC + 5 and PVC–PVC + 5 groups. Meanwhile, the upregulated gene *abcg8* was linked to ABC transporter, and the downregulated gene *agxta* was linked to peroxisome. However, the genes involved in the peroxisome were different from those in the control–PVC group, like *ech1* and *pradx1*.

### 3.4. The Gene Expression Levels of Zebrafish Larvae Affected by MPs or MPs and METH

**Figure 5:**
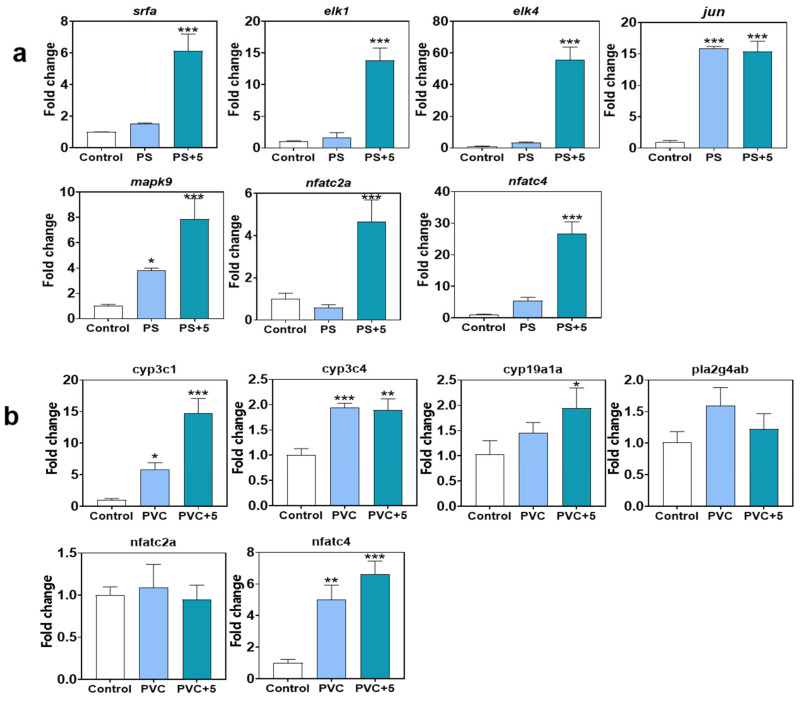
The gene expression levels of zebrafish larvae affected by MPs or MPs and METH. Gene expression levels of zebrafish juveniles in the PS groups (**a**). Gene expression levels of zebrafish juveniles in the PVC groups (**b**). All the data are expressed as mean ± S.D. * *p* < 0.05, ** *p* < 0.01, *** *p* < 0.001 vs. controls; post hoc Tukey’s test for significant ANOVA data.

Based on the RNA sequencing results of zebrafish larvae, the genes *srfa*, *elk1*, and *elk2* involved in the MAPK signaling pathway, *jun* and *mapk9* involved in apoptosis pathways, and *nfatc2a* and *nfatc4* involved in the C-type lectin signaling pathway in the PS groups were quantitatively analyzed (Figure 5a). Genes *srfa*, *elk1*, and *elk2* were significantly upregulated by PS + METH at 5 mg/L (*p* < 0.05) with 6.1-fold, 13.8-fold, and 55.8-fold, respectively, while individual exposure to PS showed no effects on the expression levels (*p* > 0.05 vs. the control). However, the significant upregulation of gene *jun* was found both in the PS and PS + 5 groups (*p* < 0.05, 15.8-fold and 15.4-fold). The increased transcriptional levels of *mapk9* were shown in the PS and PS + 5 groups with dose–response change (*p* < 0.05, 3.8-fold and 7.9-fold). Meanwhile, the expression levels of the genes *nfatc2a* and *nfatc4* were stimulated by combined exposure of PS and METH at 5 mg/L (4.6-fold and 26.7-fold, respectively).

Meanwhile, the genes *cyp3c1*, *cyp3c4*, and *cyp19a1a* (involved in steroid hormone biosynthesis pathways), *pla2g4ab* (involved in the glycerophospholipid metabolism pathway), and *nfatc2a* and *nfatc4* (involved in the C-type lectin signaling pathway) of zebrafish larvae in PVC groups were quantitatively analyzed (Figure 5a). Genes, *cyp3c1*, *cyp3c4*, and *cyp19a1a* were all upregulated in the PVC and PVC + 5 groups, and were significantly upregulated by PVC + METH at 5 mg/L (*p* < 0.05) with 14.7-fold, 1.9-fold, and 1.9-fold, respectively. However, the genes *pla2g4ab* and *nfatc2a* showed no effects on the expression levels (*p* > 0.05 vs. the control) in the PVC and PVC + 5 groups. Moreover, significant upregulation of *nfatc4* was found in the PVC and PVC + 5 groups (*p* < 0.05, 4.9-fold and 6.6-fold).

## 4. Discussion

In this study, the effects on the ecology-associated indicators of zebrafish larvae of the primary MPs (PS and PVC) were estimated. Previous studies have indicated the harmful effects of MPs on fish, including oxidative stress, intestinal damage, aberrant behavior functions, disruption development, and morality in fish [51,52]. Similarly, significantly higher mortality of larvae was found in the exposure groups (PS and PVC) than in the control. Since the survival rate of fish larvae is important for the population’s prosperity [52], exposure to PS or PVC might threaten fish flourishment. The histopathological results showed significant depositions of PS and PVC MPs in the digestive tract of fish larvae, which might contribute to MP exposure [53]. Noticeably, the depositions of PVC MPs in the intestinal tracts of zebrafish in the combined exposure groups were much greater than in the PVC single exposure group, indicating that the presence of METH might facilitate the ingestion of PVC MPs by fish. When exposure was combined with METH, the lethal effects of PS + METH at 5 mg/L significantly enhances, suggesting the joint toxicity of the two pollutants. Similarly, Qu et al. (2020) found that METH could be significantly absorbed by PS MPs at 20 mg/L, and the acute toxicity (LC50) to the zooplankton increased upon the co-contamination of PS and METH [41]. However, the addition of METH did not change the lethal effects of PVC, which was consistent with a previous study on PVC and di(2-ethylhexyl) phthalate [54]. The toxic effects of MPs on aquatic vertebrates were mainly associated with oxidative damage [52] and thus induced inflammation in fish [55]. Moreover, METH exposure at low concentrations might induce oxidative stress and morphological developmental abnormalities in zebrafish [56]. Therefore, it should be determined whether the co-exposure of MPs (PS and PVC) and METH enhances the toxicity to zebrafish. The histopathological changes in zebrafish larvae demonstrated that the presence of methamphetamine exacerbates the harmful effects of microplastics on fish, including enlarged trunk development and intestinal hydrops, but there are some differences in the pathological changes observed in the larvae among different microplastics groups, suggesting the different toxicity of varying types of MPs [57].

Since fish are the top predators of aquatic ecosystems, changes in their ecology-associated behaviors are considered to be important biomarkers for evaluating MP toxicity [58]. A previous study has evidenced the neurotoxic effect of PS and PVC MPs on fish, showing inhibition of locomotion [59]. In this study, the same results were found in zebrafish larvae, including the simplified swimming trajectory, extended immobility, and lower mean velocity. Noticeably, exposure to METH showed the same anesthetic effects on zebrafish [56]. The addition of METH significantly alleviated the effects on the immobility duration of larvae induced by PS MPs while having no impact on the PVC group. Meanwhile, the turn angle decreased exposure to PS and PVC MPs, which was then mitigated with combined exposure to METH. The results indicated that the combined exposure with METH might mitigate the adverse effects of MPs on behavioral functions of zebrafish larvae, and showed differences between PS and PVC MPs. From an ecological perspective, fish rely on swimming behavior for predation and avoiding predators in water, and reduced movement activity can diminish their success rates in these activities [60], indirectly leading to increased mortality rates and severe ecological consequences.

At this stage, studies on the toxic mechanisms of MPs on aquatic organisms have found that lipid metabolism and energy metabolism of zebrafish are suppressed by long-term PS MP stress [51]. Exposure to PVC MPs stimulated the lipid metabolic pathway and the phosphoinositide 3 kinase (PI3K)/protein kinase B (Akt) signaling pathway in mice liver [61]. However, the different mechanisms of the PS and PVC MPs, as well as combined exposure with METH, were not determined. Hence, this study for the first time elucidated the underlying mechanisms based on RNA seq. The results revealed that the majority of significantly differentially expressed genes were closely associated with metabolic pathways, such as drug metabolism, glycerolipid metabolism, and steroid synthesis. Therefore, it can be inferred that both single and combined exposures might interfere with metabolic functions in zebrafish larvae. Previous research has indicated that disruptions in fish metabolic functions can impact various aspects of fish health, behavioral traits, and survival rates [62]. The outcomes are consistent with the experimental results of this study, where significant differences were observed in mortality rates, swimming behavior, and histopathological slices of zebrafish larvae exposed solely or jointly to microplastics compared to the control group.

Furthermore, we quantitatively analyzed the expression levels of the genes involved in the MAPK signaling pathway and the C-type lectin signaling pathway of fish in the PS groups. The results indicated that the activation of the MAPK signaling pathway and the C-type lectin pathway in zebrafish larvae might contribute to the adverse effects on their ecology-associated indicators (e.g., mortality and histopathology) induced by a combination of PS and METH. The mitogen-activated protein kinase (MAPK) family consists of a group of serine/threonine kinases that mediate intracellular signal transduction [63]. The MAPK pathways are activated by various extracellular and intracellular stimuli, including peptide growth factors, cytokines, hormones, and various cellular stressors such as oxidative stress and endoplasmic reticulum stress [62]. Hence, the upregulation of the genes (i.e., *srfa*, *elk1*, and *elk2*) of fish observed in the PS + METH group indicated that combined exposure with PS MPs and METH could disrupt the normal physiological activities of cells, thereby influencing the growth and development of the larvae through activating the MAPK signaling pathway [62]. Given that the upregulation of mRNA expression in the genes *jun* and *mapk9*, related to the apoptotic pathway, can promote cell apoptosis [64], the significant increase found in this study (PS and PS + 5) demonstrated that cell apoptosis might contribute to the adverse effects induced by single or joint exposure to PS MPs and METH. C-type lectins are important pattern recognition receptors in the immune system, and the normal expression of genes in the c-type lectin signaling pathway is crucial for the immune system [65]. Genes related to the c-type lectin signaling pathway, *nfatc2a* and *nfatc4*, were significantly upregulated by PS + METH implied that combined exposure induced immune responses of zebrafish larvae. Following the impairment of the normal immune system in juvenile fish, it might lead to increased susceptibility to infections, growth limitations, decreased survival capabilities, and adverse impacts on ecological balance [66].

Meanwhile, we quantitatively analyzed the expression levels of the genes involved into steroid hormone biosynthesis pathways and C-type lectin signaling pathway of fish in PVC groups. The results implied that the combined exposure of PVC and METH might lead to adverse effects on ecology-associated indicators (i.e., mortality and behavioral functions) in zebrafish larvae through the activation of steroid hormone biosynthesis pathways and the C-type lectin signaling pathway. Cytochrome P450 (CYP) enzymes are among the most important metabolic enzymes in the liver. They are involved in the metabolism of endogenous compounds (such as steroids) and exogenous compounds (such as drugs, carcinogens, and toxic substances). Many studies have indicated that the accumulation of these exogenous compounds in the body can induce changes in gene expression and enzyme function in CYP enzymes, leading to a decrease in their pharmacological effects or toxicity [67]. The genes *cyp3c1*, *cyp3c4*, and *cyp19a1a* are involved in the synthesis of these CYP enzymes. Hence, the upregulation of these genes of fish observed in the PVC + METH group indicated that combined exposure with PVC MPs and METH could affect larvae normal liver metabolism, thereby influencing the growth and development of the larvae through changing the steroid hormone biosynthesis pathways [67]. The gene *nfatc4*, related to the C-type lectin signaling pathway, was significantly upregulated by PVC + METH, implying that combined exposure induced immune responses in zebrafish larvae. When zebrafish larvae experience a compromised immune system, it can result in heightened vulnerability to infections, growth constraints, diminished survival abilities, and detrimental effects on ecological equilibrium [66]. As such, the significant upregulation of gene mRNA expression in the combined exposure group suggested that the combined toxicity of PS + METH or PVC + METH was likely to have a substantial impact on the growth and development of zebrafish larvae, which should be a cause for concern.

## 5. Conclusions

In summary, this study reveals the combined toxicity of PS and PVC MPs and METH in zebrafish larvae. The results demonstrate increased larval mortality, abnormal behavioral patterns, and histopathological alterations upon exposure to PS and PVC microplastics. The addition of METH might enhance these adverse effects. Meanwhile, transcriptomic data showed that exposure to PS and METH activated MAPK signaling pathways and the C-type lectin signaling pathway, and PVC and METH stimulated steroid hormone synthesis pathways and the C-type lectin signaling pathway, which might then trigger apoptosis and immune responses in fish. This study for the first time elucidated the joint toxicity of primary MPs (PS and PVC) and the illicit drugs (METH) to zebrafish larvae, and the underlying mechanisms were investigated. The results should promote the development of ways of preserving the environment and protecting public health from risks associated with the co-pollution of MPs and pharmaceuticals.

## Data Availability

Data are contained within the article.

## Supplementary Materials

The following supporting information can be downloaded at: https://www.mdpi.com/article/10.3390/toxics12010064/s1, Figure S1: title; Table S1: Primer sequences for the genes tested in the present study; Figure S1: Purity of PS and PVC microplastics identified by Fourier transform infrared spectroscopy; Figure S2: Electron microscope scans and particle size of PS and PVC microplastics, Monomer of PS (a–c) and PVC (d–f) MPs showed as smooth and intact sphere; Figure S3: The correlation networks of PS or PS and METH based on the DEGs and the enrichment pathways; Figure S4: The correlation networks of PVC or PVC and METH based on the DEGs and the enrichment pathways.
