# Supplementary material for: The Exploration of Joint Toxicity and Associated Mechanisms of Primary Microplastics and Methamphetamine in Zebrafish Larvae
Source: Toxics. 2024 Jan 12;12(1):64. doi: 10.3390/toxics12010064 (PMC10820113; doi:10.3390/toxics12010064)
Supplement: Supplementary file 1 [file toxics-12-00064-s001.zip › Supporting Information toxics-2802592.pdf]

Table S1. Primer sequences for the genes tested in the present study

Genes name	Primer name	Primer sequence (5’-3’)	Genbank No.
srfa	Forward	CATACAGTGTCCACAGACGCA	NM_001110526.1
	Reverse	CTGGGATGAACCTGGATGGC	
elk1	Forward	CTCTGGCGTCTCGTCAAGTT	XM_691202.7
	Reverse	TGTAAACAACCCATCCGGGG	
elk4	Forward	GCTCAATCACCTCTGGCTGA	NM_001308956.1
	Reverse	CATCAGAAAGTGTCGTACCTGC	
jun	Forward	CATGAACCACGTCAACAGCG	NM_199987.1
	Reverse	CGAGTGAAGTTTTGCGCTCC	
mapk9	Forward	CCATGCAAAGAGGGCCTACA	XM_001919653.6
	Reverse	CCTGGAACTCCTCCAACGAC	
nfatc2a	Forward	AAGCCCTGAGTCTCCGACA	XM_021470032.1
	Reverse	GGAGTATCAAGAGCGGCTTCC	
nfatc4	Forward	AGTCAGGAAAGCATCCAGGC	XM_685091.8
	Reverse	CCGAAGCTCAATGTCGGAGT	
cyp3c1	Forward	GAGCGGTGGTGAGCATTAGT	NM_212673.1
	Reverse	TGGCAAAGGTCTTGGTCCTG	
cyp3c4	Forward	ACGGTGGTGATCAATGGCTT	NM_001077548.1
	Reverse	GGCCCGAGTCCAAAAGGTAT	
cyp19a1a	Forward	GACTGGCTGCACAAAAAGCA	NM_131154.3
	Reverse	CAAGTTTCTCTGCGTGTGCC	

The detailed protocols of RNA extraction, sequencing and analysis

A total amount of 1μg RNA per sample was used as input material for the RNA sample preparations. Sequencing libraries were generated using NEBNext® UltraTM RNA Library Prep Kit for Illumina® (NEB, USA) following manufacturer's recommendations and index codes were added to attribute sequences to each sample. The clustering of the index-coded samples was performed on a cBot Cluster Generation System using TruSeq PE Cluster Kit v3-cBot-HS (Illumia) according to the manufacturer's instructions. After cluster generation, the library preparations were sequenced on an Illumina Novaseq platform and 150 bp paired-end reads were generated. Paired-end clean reads were aligned to the reference genome using Hisat2 v2.0.5. The gene expression level was quantified by featureCounts v1.5.0-p3. Then, differential expression of Exposure and Control group was analyzed using the DESeq2 R package (1.16.1). The resulting P-values were adjusted using the Benjamini and Hochberg's approach for controlling the false discovery rate. Genes with an adjusted P-value <0.05 found by DESeq2 were assigned as differentially expressed.

The detailed protocols of quantitative real-time PCR analysis

1. Prepare the following mixture in the qPCR tube

2 × miRNA Universal SYBR qPCR Master Mix	10.0 µl
Specific Primer (10 µM)	0.4 µl
mQ Primer R (10 µM)*	0.4 µl
Template DNA/cDNA	x µl
ddH2O	To 20.0 µl

2. Perform qPCR reaction according to the following conditions

Stage 1	Pre-denaturation	Reps: 1	95℃	5 min
Stage 2	Circular reaction	Reps: 40	95℃	10 sec
			60℃	30 sec
Stage 3	Melting curve	Reps: 1		15 sec
				60 sec
				15 sec

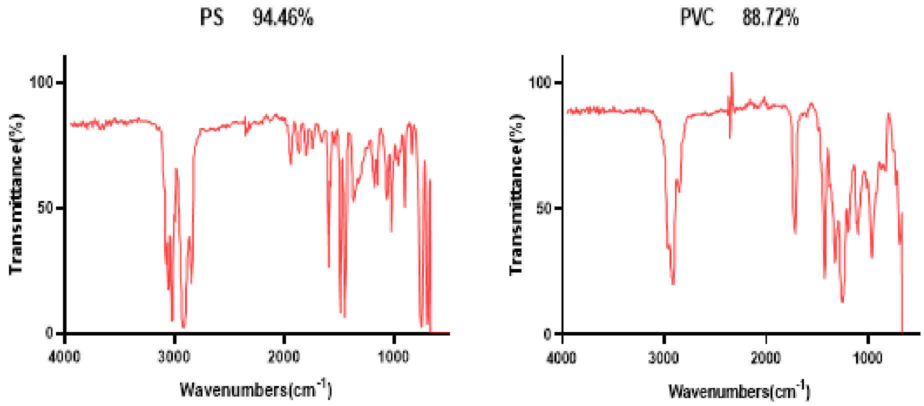

Figure S1. Purity of PS and PVC microplastics identified by Fourier transform infrared spectroscopy.

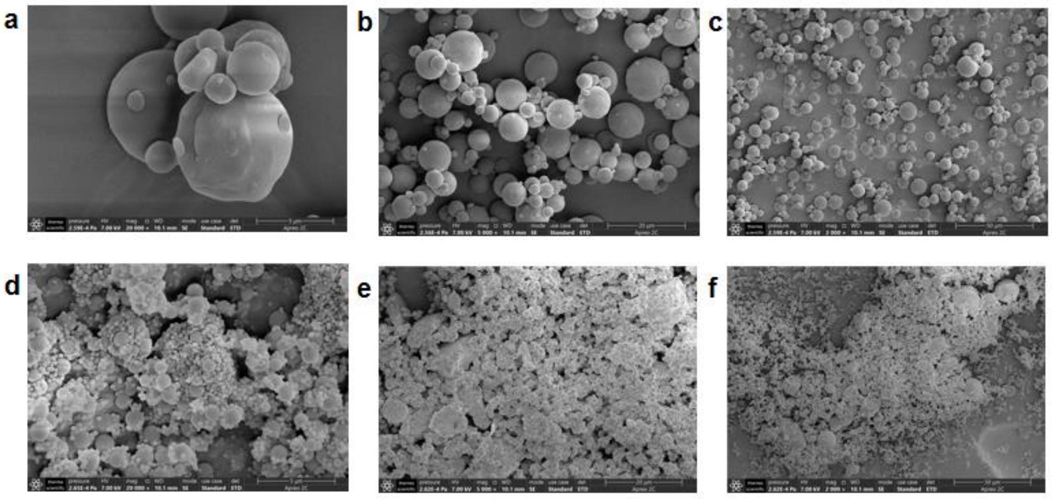

	Weighted Mean D[4,3]
PS	6.975 μm
PVC	3.760 μm

Figure S2. Electron microscope scans and particle size of PS and PVC microplastics, Monomer of PS (a-c) and PVC (d-f) MPs showed as smooth and intact sphere.

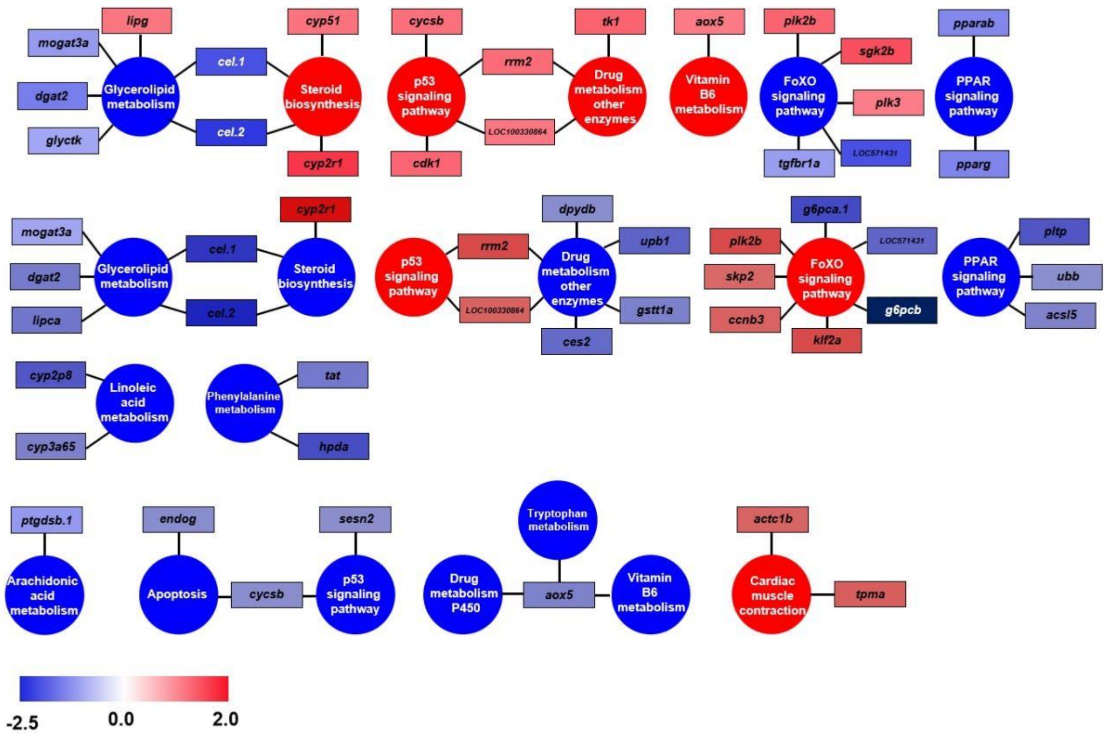

Figure S3. The correlation networks of PS or PS and METH based on the DEGs and the enrichment pathways.

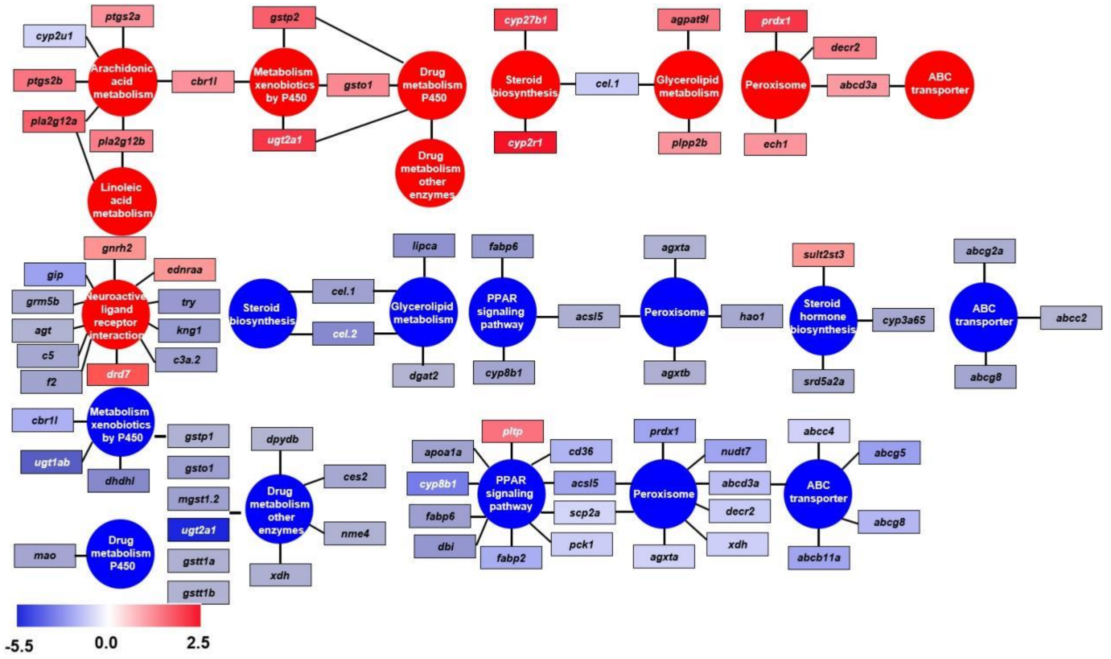

Figure S4. The correlation networks of PVC or PVC and METH based on the DEGs and the enrichment pathways.
